# Human cerebrospinal fluid extracellular vesicle miRNAs identify sex‐dependent changes in synaptic proteins in postmortem Alzheimer’s disease brain

**DOI:** 10.1002/alz.094841

**Published:** 2025-01-09

**Authors:** Ursula S Sandau, Trevor J. McFarland, Naly Setthavongsack, Sierra J Smith, Douglas R. Galasko, Joseph F Quinn, Randy L Woltjer, Julie A Saugstad

**Affiliations:** ^1^ Oregon Health & Science University, Portland, OR USA; ^2^ University of California, San Diego, La Jolla, CA USA

## Abstract

**Background:**

Alzheimer’s disease (AD) is the fifth leading cause of death for individuals aged 65 and older. Since FDA approved therapies have limited efficacy, there is a need to develop new treatments based on a better understand of pathological changes occurring in tissues from humans with AD. Central nervous system cells utilize extracellular vesicles (EVs) to package and secrete miRNAs where they mediate cell‐to‐cell communication. Importantly, cerebrospinal fluid (CSF) contains EVs derived from the brain whose contents – including miRNAs – reflect the originating cell’s molecular composition. Thus, we used CSF EV miRNAs to identify neurologically relevant genes and canonical pathways altered in the human AD brain.

**Method:**

Human CSF from AD (n = 28) and controls (n = 28) was fractionated by size exclusion chromatography to separate EVs. CSF EV miRNAs were assayed by qPCR and used for target prediction and Ingenuity Pathway Analysis. Human postmortem hippocampus from AD (n = 34) and control (n = 22) were used to measure SNAP‐25, MUNC18‐1, and Reelin by immunoblot. Human hippocampal sections were co‐labeled for SNAP‐25 and miR‐16‐5p in AD (n = 4) and control (n = 3). MiR‐16‐5p puncta were analyzed using Zen Intellesis AI Segmentation software.

**Result:**

Four miRNAs (miR‐16‐5p, ‐331‐3p, ‐409‐3p, ‐454‐3p) were significantly increased in AD CSF EVs compared to control EVs. Further, the increase in miR‐16‐5p and ‐331‐3p occurred only in AD females with APOEe3/e4 genotype. Conversely in males, miR‐16‐5p and ‐331‐3p showed no group differences. The predicted targets of miR‐16‐5p include mRNAs integral to synaptic transmission (SNAP‐25, MUNC18‐1, Reelin), which were significantly decreased in the hippocampus of AD females, compared to control females. While in males these protein targets were not changed in AD. Co‐labeling of human brain sections demonstrated that miR‐16‐5p and SNAP‐25 are both expressed in cortical grey matter and preliminary analysis shows a trend of increased miR‐16‐5p in AD.

**Conclusion:**

We demonstrate that the miRNA cargo of CSF EVs is impacted by AD, sex, and APOE4 status. Also, CSF EV miRNAs have the potential to identify therapeutic targets to treat or cure AD. These data also demonstrate pathological changes in AD are sex‐ and genotype‐dependent, which may need to be considered for drug development and/or treatment strategies.

